# A solid-phase extraction method for rapidly determining the adsorption coefficient of pharmaceuticals in sewage sludge

**DOI:** 10.1016/j.watres.2014.09.020

**Published:** 2014-12-15

**Authors:** Laurence Berthod, Gary Roberts, David C. Whitley, Alan Sharpe, Graham A. Mills

**Affiliations:** a AstraZeneca Brixham Environmental Laboratory, Freshwater Quarry, Brixham, Devon TQ5 8BA, UK; b School of Pharmacy and Biomedical Sciences, University of Portsmouth, Portsmouth, Hampshire PO1 2DT, UK

**Keywords:** Adsorption coefficient, Pharmaceuticals, Sewage sludge, Solid-phase extraction

## Abstract

The partitioning of pharmaceuticals in the environment can be assessed by measuring their adsorption coefficients (*K*_*d*_) between aqueous and solid phases. Measuring this coefficient in sewage sludge gives an indication of their partitioning behaviour in a wastewater treatment plant and hence contributes to an understanding of their subsequent fate. The regulatory approved method for measuring *K*_*d*_ in sewage sludge is the US Environmental Protection Agency's Office of Prevention, Pesticides and Toxic Substances (OPPTS) guideline 835.1110, which is labour intensive and time consuming. We describe an alternative method for measuring the *K*_*d*_ of pharmaceuticals in sewage sludge using a modified solid-phase extraction (SPE) technique. SPE cartridges were packed at different sludge/PTFE ratios (0.4, 6.0, 24.0 and 40.0% w/w sludge) and eluted with phosphate buffer at pH 7.4. The approach was tested initially using three pharmaceuticals (clofibric acid, diclofenac and oxytetracycline) that covered a range of *K*_*d*_ values. Subsequently, the sorption behaviour of ten further pharmaceuticals with varying physico-chemical properties was evaluated. Results from the SPE method were comparable to those of the OPPTS test, with a correlation coefficient of 0.93 between the two approaches. SPE cartridges packed with sludge and PTFE were stable for up to one year; use within one month reduced variability in measurements (to a maximum of 0.6 log units). The SPE method is low-cost, easy to use and enables the rapid measurement of *K*_*d*_ values for a large number of chemicals. It can be used as an alternative to the more laborious full OPPTS test in environmental fate studies and risk assessments.

## Highlights

• We develop and validate a robust method to measure *K*_*d*_.
• Stability study of the cartridges performed up to a year.
• The new method fully correlates with the OPPTS 835.1110 regulatory-approved test.

## Graphical abstract

**Figure:**
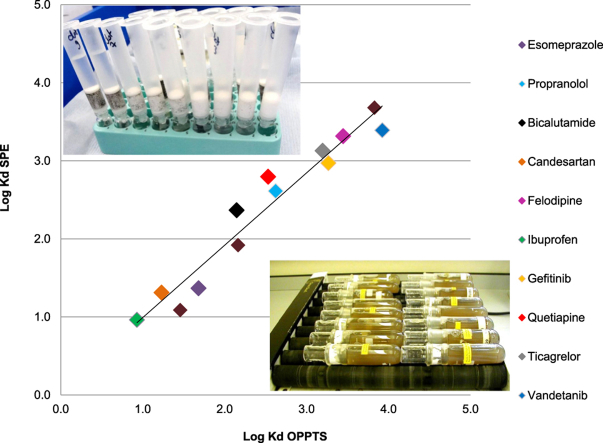

## 1 Introduction

Since 2006, all pharmaceuticals for human use in Europe require an environmental risk assessment (ERA) to be performed. In Phase II Tier A, the partitioning behaviour of active pharmaceutical ingredients (APIs) needs to be characterised (EMA, 2006). As most APIs are consumed and subsequently excreted by the patient, the main environmental exposure route is normally via a wastewater treatment plant. Therefore, the partitioning of APIs between the aqueous phase and the biosolids (sludge) present in a wastewater treatment plant is important in describing the fate of the API during this process.

The adsorption coefficient (*K*_*d*_) between water and solid (e.g. soils, sludge) phases is an important parameter in determining the environmental fate of a chemical. Many *K*_*d*_ values have been published for various pharmaceuticals including antibiotics, anticancers, anti-inflammatories, cardiovasculars, central nervous system drugs and hormones (Barron et al., 2009, Carballa et al., 2008, Göbel et al., 2005, Hörsing et al., 2011, Joss et al., 2005, Kim et al., 2010, Lajeunesse et al., 2012, Radjenović et al., 2009, Stevens-Garmon et al., 2011, Stuer-Lauridsen et al., 2000, Suárez et al., 2008, Ternes et al., 2004, Urase and Kikuta, 2005, Wick et al., 2009) for different sludge types. Over the last decade, there has been interest in determining the fate of a wide range of pharmaceuticals in wastewater treatment plants (ECHA, 2011, Schwarzenbach et al., 2003). Rogers (1996) estimated the sorption potential of hydrophobic contaminants according to their *n*-octanol/water partition coefficient (*K*_OW_) where contaminants with log *K*_OW_ < 2.5 would have a low sorption potential, those with 2.5 < log *K*_OW_ < 4.0 would have a medium sorption potential and those with log *K*_OW_ > 4.0 would have a high sorption potential. These log *K*_OW_ values may be transposed to sludge-water partition coefficients using equations such as log *K*_*d*_ = 1.14 + 0.58 × log *K*_OW_ (Dobbs et al., 1989). Chemicals with a low *K*_*d*_ value (around log *K*_*d*_ < 2.6), will remain mainly in the aqueous effluent, and those with a high *K*_*d*_ value (around log *K*_*d*_ > 3.6) will be predominantly adsorbed by the biosolids (sludge) phase. In the latter case, precautions may be required for the disposal of sludge to agricultural land, as there are potential risks associated with leaching of desorbed chemicals and the movement of these chemicals into crops destined for livestock or human consumption. Often, an organic carbon normalised version of *K*_*d*_ is used, the organic carbon partition coefficient, *K*_OC_, which is obtained from *K*_*d*_ = *K*_OC_ × *f*_OC_, where *f*_OC_ is the fraction of organic carbon in the sludge or soil (Schwarzenbach et al., 2003). APIs with log *K*_OC_ > 4 require the performance of terrestrial risk assessment (EMA, 2006).

*K*_*d*_ can be measured experimentally or estimated by mathematical models. In most instances, experimental measurements are based on a number of guidelines, e.g. Organisation for Economic Co-operation and Development (OECD) 106 (OECD, 2000), OECD 121 (OECD, 2001) or the Environmental Protection Agency's Office of Prevention, Pesticides and Toxic Substances (OPPTS) guideline 835.1110 (EPA, 1998). The OPPTS 835.1110 guideline is currently the only one that is specifically focused on partitioning behaviour in sewage sludge. In this guideline, a series of experiments are performed using different aqueous concentrations of sludge and the linear portion of the slope of the resultant isotherm is used to calculate *K*_*d*_. The method is fastidious and complex, especially for chemicals that are poorly water soluble, adsorb to the test vessels or degrade rapidly in the test. The test can take several days to complete, due to long equilibration times, phase separation and long analytical run times. Recent progress in extraction and instrumental techniques has allowed alternative methods to be used to experimentally measure *K*_*d*_ by analysing both the biosolid sludge sample and its associated aqueous supernatant (Göbel et al., 2005, Joss et al., 2005, Lajeunesse et al., 2012, Radjenović et al., 2009). Systems include stirred bottles (Hörsing et al., 2011, Wick et al., 2009) and batch type experiments in laboratory scale reactors (Ternes et al., 2004, Urase and Kikuta, 2005). All these different methods involve a certain degree of complexity, up to 14 h equilibration time and specific expensive equipment, making them not easily adaptable from laboratory to laboratory.

The mathematical modelling approach uses equations based on physico-chemical properties such as hydrophobicity (*K*_ow_), water solubility and *pK*_*a*_ (Dobbs et al., 1989, Franco et al., 2009, Franco et al., 2013, Franco and Trapp, 2008, Guo et al., 2004, Sabljic et al., 1995). Although a number of predictive equations are available, most fail to predict accurately the *K*_*d*_ value for ionisable chemicals such as many APIs. Typically the pH in the sewage sludge is 6–8. Here, basic compounds are predominantly in their molecular, uncharged form, and behave as neutrals. Acidic and Zwitterionic substances are mainly ionised and hence hydrophobicity is no longer the main mechanism of sorption. Most of the existing models are based on hydrophobicity, with only a few taking into consideration other interactions such as ion-exchange and electronegativity mechanisms.

Previously, we described a new approach to measure the *K*_*d*_ of APIs adsorbed to sewage sludge, based on a solid-phase extraction (SPE) method (Berthod et al., 2014). This initial work investigated the performance of different inert packing materials (e.g. silica, silicon carbide, polyether-ether ketone and polytetrafluoroethylene (PTFE)) that could be mixed with the sewage sludge in the SPE cartridges. PTFE was the best candidate material tested for this purpose. The objective of the current work was to use this approach to develop and validate a robust, faster alternative to the OPPTS 835.1110 test for measuring sorption of compounds to sewage sludge, in order to be able to test a larger number of chemicals in a shorter period of time.

## 2 Materials and methods

### 2.1 Chemicals and reagents

Clofibric acid (98.6%), diclofenac (99%) and oxytetracycline (97%) were obtained from Sigma Aldrich Ltd. (Gillingham, Dorset, UK). Bicalutamide, candesartan, esomeprazole, felodipine, gefitinib, ibuprofen, propranolol, quetiapine, ticagrelor and vanderanib were supplied by AstraZeneca Ltd. (Macclesfield, UK). Potassium dihydrogen orthophosphate, dipotassium hydrogen phosphate and sodium phosphate were obtained from Fisher Scientific Ltd. (Loughborough, UK). HPLC grade acetonitrile and methanol were obtained from Sigma Aldrich Ltd. All water was purified by reversed osmosis using an Elga Purelab Option-Q system (Elga LabWater, Marlow, UK).

The phosphate buffer was made by weighing 8.5 g of anhydrous potassium dihydrogen phosphate, 21.75 g of anhydrous dipotassium hydrogen phosphate and 33.4 g of disodium hydrogen phosphate dehydrate and dissolving in 1 L of water, ensuring the pH was 7.4 ± 0.5. A diluted (1:100 v/v) solution of this stock buffer was used in all experiments. This pH was chosen to match the conditions in an activation tank of a wastewater treatment plant (around pH 6–8). The phosphate buffer was made from a mixture of K^+^ and Na^+^ cations as preliminary work showed this combination was the closest to that in sludge supernatant (unpublished data).

Activated sludge was collected from Totnes wastewater treatment plant (Devon, UK) and subsequently freeze dried using a ModulyoD freeze dryer (Thermo Electron Corp., Loughborough, UK) and then oven dried at 105 °C and sieved (500 μm mesh). PTFE particles (200 μm), empty SPE cartridges (3 mL) and frits were purchased from Sigma Aldrich Ltd.

### 2.2 Sludge packed SPE cartridges

Empty SPE cartridges were packed by hand with 500 mg of each of four sludge/PTFE mixtures (0.4, 6.0, 24.0 and 40.0% w/w sludge) in triplicate to test the sorption behaviour of each individual API. Only the ratio of sludge to PTFE changed, the final weight of material packed in the cartridge remained constant at 500 mg. The four different ratios were chosen to match those in the OPPTS 835.1110 test. Sludge packing ratios above 50% w/w were avoided as they made elution through the bed difficult (Berthod et al., 2014). An additional test cartridge containing only PTFE (500 mg) was prepared. This was used to measure any sorption of the test compounds to the PTFE, plastic walls or frits of the SPE cartridge. PTFE was chosen as the bulk packing material as it is chemically inert, and was shown previously to exhibit minimal sorption for the test APIs; hence, all of the observed sorption behaviour could be attributed to the sludge (Berthod et al., 2014). Two control cartridges (one containing 500 mg of PTFE and the other packed with 500 mg of 40% w/w sludge in PTFE) were prepared to measure any background concentrations of the APIs present in either the PTFE or the sludge. These cartridges were not dosed with the test compounds (Fig. S1 in supplementary information).

### 2.3 Sample preparation

All the packed SPE cartridges were first fully hydrated with phosphate buffer (2 mL) by letting the solution freely percolate through the bed by gravity over ∼15 min. The thirteen test cartridges (whilst still moist from the previous step) were loaded with an aliquot (400 μL) of an aqueous solution (50 mg/L) of each individual API (or, in one experiment, a mixture of clofibric acid, diclofenac and oxytetracycline, all at 50 mg/L). The solution was allowed to percolate slowly into the bed (∼15 min). The APIs were then eluted using two sequential aliquots (2 mL) of phosphate buffer, being allowed to percolate freely for a time ranging from ∼30 min for the lower sludge ratio to ∼120 min for the higher ratio. This procedure ensured a high desorption efficiency for the analytes from the sorbent phase. The two sequential eluates were collected in separate vials (2 mL). The dissolved aqueous concentration of each test API contained in each vial was analysed by HPLC. The two control cartridges were not loaded with APIs, but eluted only using two sequential aliquots (2 mL) of phosphate buffer and the eluates analysed as above. The total aqueous concentration of each API was estimated as the sum of the concentration found in each of the two eluates.

### 2.4 Chromatography

An HPLC system (Agilent 1100, Agilent Technologies, Waldbronn, Germany) connected to a UV detector (200–350 nm wavelength) was used to measure the concentration of the APIs in the eluates. The column was a Gemini C_18_ (Phenomenex, Macclesfield, UK) 150 mm × 4 mm with 3 μm particles held at 40 °C. All analyses were performed with a flow rate of 0.7 mL/min, 50–140 kg/cm^2^ pressure and an injection volume of 20 μL. The APIs were separated using an acetonitrile/phosphate buffer gradient elution (15 min run time). Analysis started with a phosphate buffer (0.01 M, pH 3.0)/acetonitrile (90/10 v/v) mobile phase (1 min) followed by linear increase in acetonitrile from 10% to 70% (v/v) over 10 min. The final composition was held for 2 min before returning to the initial solvent composition. The detection wavelength was 220 nm, giving a limit of quantification for clofibric acid, diclofenac and oxytetracycline of 0.10, 0.03 and 0.10 mg/L, respectively. The detection limits for bicalutamide, candesartan, esomeprazole, felodipine, gefitinib, ibuprofen, propranolol, quetiapine, ticagrelor and vanderanib were 0.03, 0.01, 0.02, 0.01, 0.09, 0.02, 0.01, 0.03, 0.02 and 0.01 mg/L, respectively. Limits of quantification were assumed to be three times the limit of detection for each analyte. Quantification was performed using external standards containing the APIs at known concentrations (range 0.1–500 mg/L). Chromatograms were analysed using Laura software version 4.0.2.75 (LabLogic, UK).

### 2.5 Method development

The SPE method was developed using three test APIs: clofibric acid, diclofenac and oxytetracycline, representing low, medium and high *K*_*d*_ values, respectively. These compounds were chosen for their acidic or Zwitterionic character. Their physico-chemical properties are shown in Table 1.

**Table 1:** Physico-chemical properties of the three test APIs used in method development.

Compound	Clofibric acid	Diclofenac	Oxytetracycline
Structure			
Pharmaceutical class	Lipid regulator	Analgesic, anti- inflammatory	Antibacterial, antibiotic
MW	215	296	460
log *K*_ow_^a^	2.7	4.1	1.6
*pK*_*a*_	3.0	4.15	3.3, 7.3, 9.1
log *K*_*d*_	1.5^b^	1.5–2.7^b^^,^^c^	3.5^d^

a Predicted by ACD Lab (www.acdlabs.com).

b Urase and Kikuta, 2005.

c Ternes et al., 2004.

d Stuer-Lauridsen et al., 2000.

The *pK*_*a*_ values suggest that clofibric acid and diclofenac were in the neutral form at low pH, (e.g. pH 2) and in a negatively charged (carboxylate anion) form at intermediate and high pH values such as the pH of the phosphate buffer eluent (pH 7.4). Oxytetracycline was always in its charged form. At low pH its tertiary amine group was positively charged. At pH 4.5, the isoelectric point, it would be a Zwitterion (positive amine plus negative phenol) and at higher pH values such as in this study (pH 7.4), it would have a negative charge (Table 1).

APIs were tested individually following the OPPTS 835.1110 guideline and using the SPE method. A similar experiment was conducted with a solution of all three test compounds (all at 50 mg/L) to assess if potential interferences between individual APIs was likely to affect the results.

### 2.6 Method validation

In order to test the method more extensively, ten additional APIs with a range of physico-chemical properties were tested individually using the SPE and OPPTS 835.1110 methods. The ten APIs covered a wide range of *pK*_*a*_, log *K*_OW_, molecular weights and therapeutic areas (Table S1); they are widely used and hence likely to be found in wastewater treatment plants. Our laboratory had previously measured the *K*_*d*_ value (OPPTS 835.1110 guideline) of these substances.

The overall reproducibility (ruggedness) of the method was evaluated by analysing the sorption behaviour of the three test compounds with two different samples of activated sewage sludge collected (separated by a three month interval) at the Totnes wastewater treatment plant. Sets of cartridges were packed freshly and eluted using freshly prepared buffer.

### 2.7 Stability study

A stability study was conducted at the following time points: initial (T0), 1 week (T1), 1 month (T2), 3 months (T3), 6 months (T4) and 1 year (T5) to assess whether SPE cartridges could be pre-packed in bulk and subsequently stored for future use, or if they had to be packed freshly for each experiment.

### 2.8 Calculations

For each SPE cartridge, a *K*_*d*_ value was calculated using Eq. (1) based on the linear Freundlich isotherm for solution at low concentration.

(1) $$K_{d}=\frac{X/m}{C_{\text{aqueous}}}$$

Here, *C*_aqueous_ is the concentration of the API eluted from the SPE cartridge (mg/L); *X* is the mass of API adsorbed onto sludge (mg), calculated from the difference between the nominal aqueous concentration added initially to the test cartridge and *C*_aqueous_; and *m* is the dry mass of sewage sludge used (kg). A plot of *C*_aqueous_ versus *X*/*m* was made for each compound to ensure the experimental conditions remained in the linear part of the isotherm (at steady state). The overall *K*_*d*_ was calculated as the slope of the linear correlation between C_aqueous_ and *X*/*m* over all four different sludge ratios for the three replicates (12 data points). An example of the calculation of *K*_*d*_ is shown in the supplementary information (Fig. S2).

## 3 Results and discussion

### 3.1 Method development

**Fig. 1:**
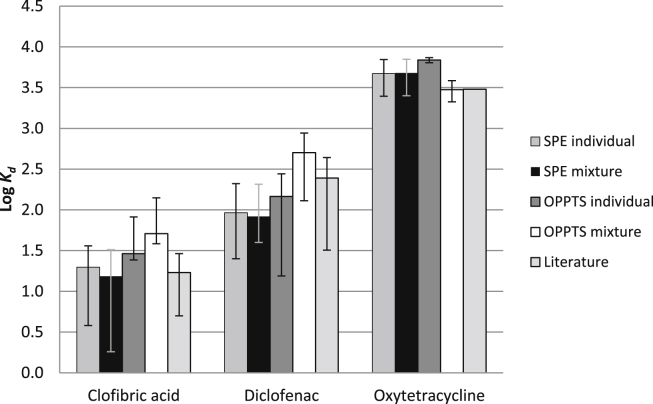
Comparison between *K*_*d*_ values (as logarithms) obtained with the SPE method and the OPPTS 835.1110 test. Values of *K*_*d*_ are given for the three APIs tested individually and as a mixture, together with their respective literature values (Table 1). The error bars represent 95% confidence intervals. Only one literature value was available for oxytetracycline, so no error bar is included for this case.

For the SPE method with the three test APIs, the *K*_*d*_ values and 95% confidence intervals (CI) were *K*_*d*_ = 20, CI = 4–36 (individual) and *K*_*d*_ = 15, CI = 2–33 (in mixture) for clofibric acid, *K*_*d*_ = 92, CI = 25–210 (individual) and *K*_*d*_ = 83, CI = 40–206 (in mixture) for diclofenac and *K*_*d*_ = 4,704, CI = 2480–6980 (individual) and *K*_*d*_ = 4,788, CI = 2516–7060 (in mixture) for oxytetracycline. These values were comparable with those found with the OPPTS 835.1110 test and with values reported in the literature. The logarithmically (log 10) transformed data (*K*_*d*_ and associated CIs) are shown in Fig. 1.

In the case of clofibric acid, the CIs shown in Fig. 1 for the SPE method are larger than those for the OPPTS test. This, however, is an artefact of the logarithmic scale. For the OPPTS test, the *K*_*d*_ values and CIs for clofibric acid were *K*_*d*_ = 29, CI = 24–82 (individual) and *K*_*d*_ = 51, CI = 38–141 (in mixture). These CIs are wider than those found with the SPE method.

A two-sided *t*-test on the *K*_*d*_ values confirmed there was no evidence for a statistically significant difference (*p*-value = 0.518, CI for the differences = −155, 107) between the sorption behaviour when the three APIs were applied to the sludge-packed SPE cartridge either individually or as a mixture. For the OPPTS 835.1110 test, the *K*_*d*_ values obtained by testing the APIs individually and in a mixture were also comparable to each other (*p*-value = 0.481, and CI = −4710, 7059), but the CIs were smaller for diclofenac, similar for clofibric acid and slightly wider for oxytetracycline when tested together in a mixture. These results indicated that the SPE method can potentially measure simultaneously, the *K*_*d*_ values of mixtures of compounds, thereby increasing laboratory throughput. None of the three APIs tested were detected in the sludge or the PTFE used in the control cartridges. The recovery of the test APIs from the cartridge containing only the PTFE support matrix was 100 ± 2% (*n* = 3). This indicated that there was no sorption of the chemicals to the PTFE or SPE cartridges. All the observed sorption could therefore be attributed to the sludge.

Although the experiment involved measuring the sorption of only three compounds, the correlation between log *K*_*d*(OPPTS)_ and log *K*_*d*(SPE)_ gave an *R*^2^ = 0.99 with an equation of log *K*_*d*(SPE)_ = 1.05 × log *K*_*d*(OPPTS)_ − 0.35. This result indicated almost a 1:1 relationship between *K*_*d*_ values obtained by the SPE and OPPTS tests (Fig. S3) and, therefore, the method was evaluated with a larger set of diverse APIs.

### 3.2 Method validation

**Fig. 2:**
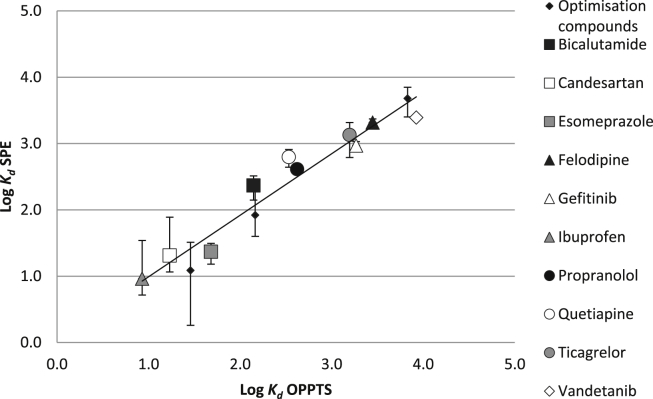
Plot of log *K*_*d*_ values obtained using the SPE and OPPTS 835.1110 methods for ten additional APIs to the three optimisation compounds (clofibric acid, diclofenac and oxytetracycline) with varying physico-chemical properties (Table 1 and S1). The solid-line is the linear fit to the data: *y* = 0.93*x* + 0.06 (*R*^2^ = 0.94, *n* = 13). The error bars show the 95% confidence intervals for log *K*_*d*_ obtained with the SPE method.

To further validate the method, ten additional APIs were tested. *K*_*d*_ values were obtained using the SPE method and compared with those obtained with the OPPTS 835.1110 test. A log–log plot of these data (together with the results for the three test APIs) gave a linear relationship with slope = 0.93 and *R*^2^ = 0.94 (Fig. 2). No APIs were detected in the sludge used in the control cartridges. Recovery of the ten APIs from the cartridges containing only PTFE was 100 ± 2% (*n* = 10).

The data show that *K*_*d*_ values obtained with the SPE method were comparable to values obtained with the OPPTS 835.1110 test. Repeatability of the SPE method was assessed by retesting the three test APIs using two samples of activated sewage sludge collected from the same wastewater treatment plant separated by a three-month period. The variability of the *K*_*d*_ values is shown in Table 2. This range of variability is similar to that found with the OPPTS 835.1110 test due to the heterogeneous nature of the sewage sludge. For the OPPTS 835.1110 test a maximum variability of *K*_*d*_ within a factor of 2 is considered acceptable, which corresponds to a range of 0.6 log units. Using the SPE method, the maximum variability was 0.16 log units, observed for diclofenac.

**Table 2:** Variability, over a three-month period in the measurement^a^ of *K*_*d*_ for three test APIs using the SPE method.

APIs	Test 1		Test 2	
	*K*_*d*_	log *K*_*d*_	*K*_*d*_	log *K*_*d*_
Clofibric acid	18.3	1.26	18.2	1.26
Diclofenac	344.5	2.54	238.8	2.38
Oxytetracycline	6056.0	3.78	4542.4	3.66

a Measurements based on for four sludge/PTFE mixtures (0.4, 6.0, 24.0 and 40.0% w/w sludge) performed in triplicate for each test API.

### 3.3 Stability study

**Fig. 3:**
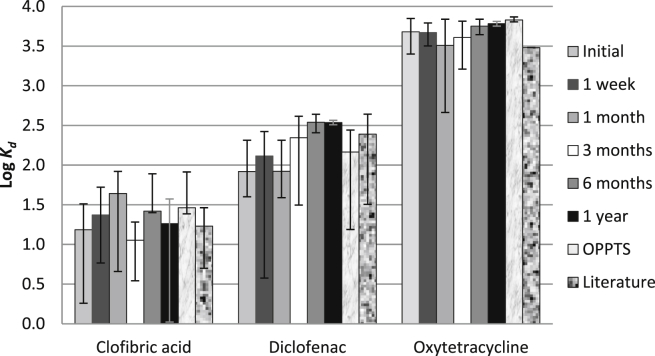
Plot of log *K*_*d*_ values obtained using the SPE method at six time points and OPPTS 835.1110 test, together with their respective literature values. The error bars represent 95% confidence intervals.

A twelve month stability study, at six defined time points was performed to establish the shelf-life of the prepared SPE cartridges. The SPE cartridges showed good stability over time (Fig. 3). The variability in *K*_*d*_ was less than 0.5 log units for clofibric acid and less than 0.3 log units for oxytetracycline. Diclofenac had the highest variability (0.62 log units) between 1 month and 6 months, which indicates that cartridges were best used within a month, but were potentially usable for up to a year. The sorption of diclofenac is known to be variable, and is evidenced by the large range of *K*_*d*_ values reported in the literature (*K*_*d*_ = 32–459). This variability was within a factor of two of the OPPTS 835.1110 test results (diclofenac *K*_*d*_ = 146, CI = 15–277, variability of 1.27 log units).

### 3.4 Comparison of resource requirements of the SPE and OPPTS 835.1110 methods

The OPPTS 835.1110 guideline recommends a sample equilibration time of 16 h. In addition, samples require a centrifugation step to separate biosolids from the aqueous phase prior to chemical analysis. With the SPE method, the set of 15 cartridges could be eluted within 4 h. This shorter analysis time limits the possibility of biodegradability, with no need for sterilisation of the sludge or the equipment or special aseptic sampling handling procedures.

## 4 Conclusion

We have developed an alternative, simple, rapid and low-cost method for measuring *K*_*d*_ values of APIs in sewage sludge. This method gave comparable *K*_*d*_ values to those obtained using the OPPTS 835.1110 guideline. The new method is potentially applicable for use in environmental risk assessments, enabling more economic, wider-scale testing of a diverse range of compound classes. The stability of the packed SPE cartridges was found to be satisfactory for up to a year. However, in order to reduce variability it is recommended the cartridges are used within a month of packing.

While the method was validated using activated sludge, it may be also possible to use other types of biosolids (e.g. primary, secondary or digested sludge) found within a wastewater treatment plant. Our approach of using packed SPE cartridges to measure *K*_*d*_ could also be extended to other solid environmental matrices, such as soils and sediments, however, this would require further work to assess the validity of the method in those contexts.
