# Supplementary material for: A solid-phase extraction method for rapidly determining the adsorption coefficient of pharmaceuticals in sewage sludge
Source: Water Res. 2014 Dec 15;67:292–8. doi: 10.1016/j.watres.2014.09.020 (PMC4234481; doi:10.1016/j.watres.2014.09.020)
Supplement: Supplementary file 1 [file mmc1.docx]

**Supplementary Information**

# A solid-phase extraction method for rapidly determining the adsorption coefficient of pharmaceuticals in sewage sludge

# Laurence Berthod^a,b,^*^,1^, Gary Roberts^a,2^, David C. Whitley^b^, Alan Sharpe^a,1^, Graham A. Mills^b^

*^a^AstraZeneca Brixham Environmental Laboratory, Freshwater Quarry, Brixham, Devon, TQ5 8BA, UK*

*^b^School of Pharmacy and Biomedical Sciences, University of Portsmouth, Portsmouth, Hampshire, PO1 2DT, UK*

*Corresponding author. Tel.: [+44 (0) 1795441553](tel:%2B44%280%291803884298)

E-mail address: [laurence.berthod@port.ac.uk](mailto:laurence.berthod@port.ac.uk)

*^1^* Current address: *AstraZeneca UK Ltd, Alderley Park, Macclesfield, SK108TG*

*^2^* Current address: *ENVIRON UK Ltd, Aston Court, Pynes Hill, Exeter, EX2 5AZ, UK*

**Table S1**: Physico-chemical properties of the ten APIs used in method validation. Data reported on the AstraZeneca portal**^a^**, with the exception of the *K_d_* value for ibuprofen, which is from an internal AstraZeneca report (unpublished data). All *K_d_* values were obtained using the OPPTS 835.1110 test.

| **Compound** | **Structure** | **Therapeutic area** | **MW** | **log *K_OW_*** | ***pK_a_*** | ***K_d_*** |
| --- | --- | --- | --- | --- | --- | --- |
| Bicalutamide | 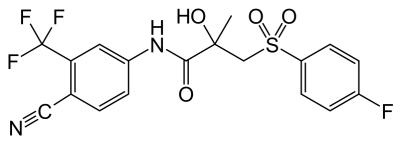 | Non-steroidal anti-androgen | 430.37 | 2.4 | 11.9,5 | 140 |
| Candesartan | 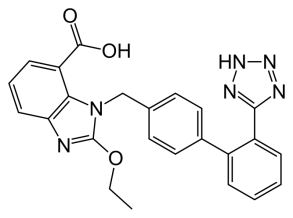 | Angiotensin receptor blockers, hypertension | 440.45 | 1.63 | 2.1, 4.6 | 17 |
| Esomeprazole | 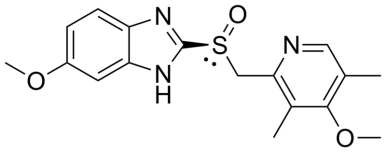 | Gastrointestinal | 345.42 | 1.65 | 4, 8.8 | 48 |
| Felodipine | 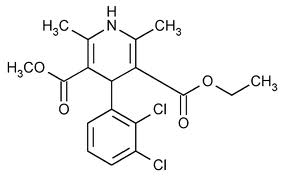 | Cardiovascular and diabetes | 384.25 | 5.0 | 5.4 | 2,800 |
| Gefitinib | 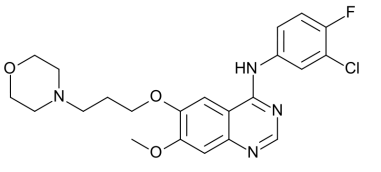 | Oncology | 446.90 | 4.15 | - | 1,852 |
| Ibuprofen | 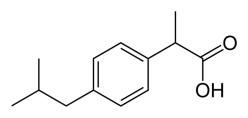 | Anti-inflammatory | 206.29 | 3.72 | 4.8 | 8.5^b^ |
| Propranolol | 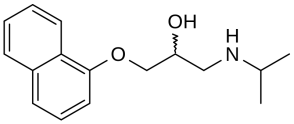 | Beta-blockers, hypertension | 259.34 | 3.48 | 9.5 | 420 |
| Quetiapine | 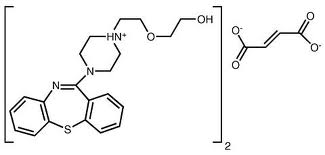 | Neuroscience | 383.51 | 1.4−2.7 | 3.3, 6.8 | 340 |
| Ticagrelor | 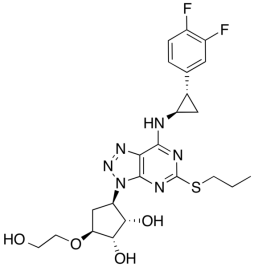 | Cardiovascular and diabetes | 522.57 | > 4.02 | - | 1,571 |
| Vandetanib | 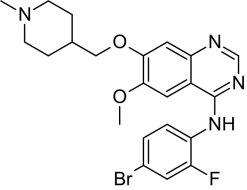 | Oncology | 475.35 | -0.7−3.9 | 9.3 | 8,400 |

^a^http://www.astrazeneca.com/Responsibility/The-environment/Pharmaceuticals-in-the-environment/era-data-2012.


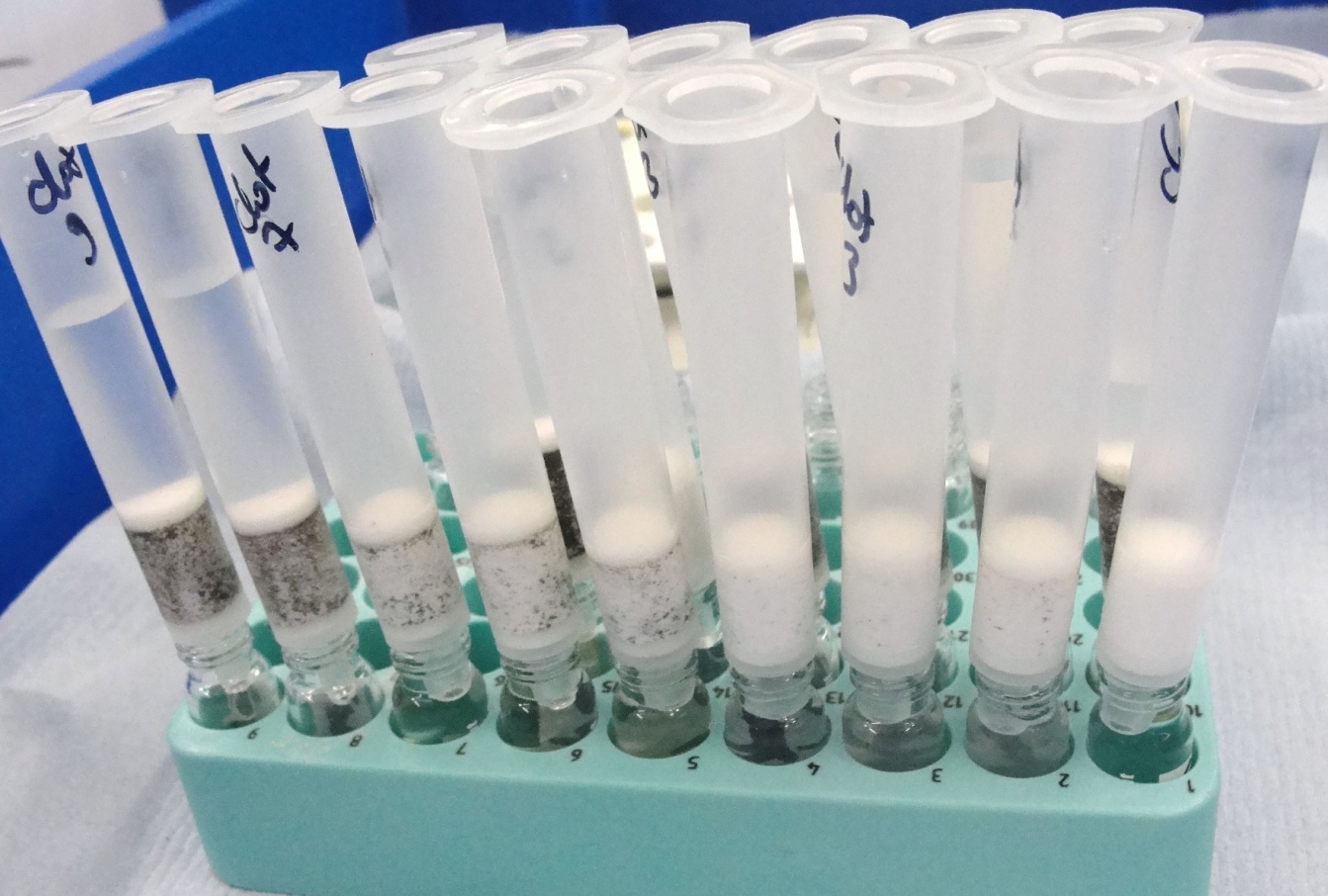


**Fig. S1**: Set-up for the fifteen cartridges used in the SPE method to determine *K_d_* values of the APIs in sewage sludge. Thirteen test cartridges were used: twelve (three replicates) were packed with four different sludge/PTFE ratios and one containing only PTFE to measure any sorption of the APIs to the apparatus. The two control cartridges, containing only PTFE and sewage sludge, were used to measure any background concentrations of the APIs.

**Fig. S2**: Plot of C*_aqueous_* (mg/L) versus *X/m* (mg/kg) values obtained using the SPE method for vanderanib. The solid line is the linear fit to the data passing through the origin: $y=2464 x (R^{2}=0.93, n=12)$. The 95% confidence interval for the slope *K_d_* is [2222, 2706].

**Fig. S3.** Plot of log *K_d_* values obtained using the SPE and OPPTS 835.1110 methods for the three test APIs (clofibric acid, diclofenac and oxytetracycline). The linear fit to the data is $y=1.05x-0.35 (R^{2}=0.99, n=3)$. The error bars represent the 95% confidence intervals.
